# Expression pattern and regulatory effect of lysine-specific demethylase 2A gene in clear cell renal cell carcinoma

**DOI:** 10.1186/s12894-021-00867-8

**Published:** 2021-08-14

**Authors:** Jiannan Wang, Tan Li, Xiang Li, Yixia Zhang, Xuemei Wang

**Affiliations:** 1 Department of Ultrasound, The First Hospital of China Medical University, No. 155 NanjingBei Street, Heping District, Shenyang, 110001 Liaoning Province People’s Republic of China; 2 Department of Cardiovascular Ultrasound, The First Hospital of China Medical University, Shenyang, 110001 People’s Republic of China

**Keywords:** Lysine-specific demethylase 2A, Renal cell carcinoma, Expression, Cell biological behavior, Survival rate

## Abstract

**Background:**

Our study aimed to explore the expression and the biological role of lysine-specific demethylase 2A (KDM2A) in clear cell renal cell carcinoma (ccRCC).

**Methods:**

In vitro, KDM2A expression was measured by qRT-PCR and western blot. A total of 50 patients with ccRCC were included, and KDM2A expression in ccRCC tissues was assessed by qRT-PCR and immunohistochemistry. The effects of KDM2A expression on cell biological behavior were examined by cell counting kit-8 assay, transwell assay and flow cytometry, respectively. The prognostic value of KDM2A in ccRCC was evaluated by Kaplan–Meier method.

**Results:**

The KDM2A expression was significantly upregulated in ccRCC cell line (*P* < 0.05). Compared with para cancer tissues, ccRCC samples showed a higher KMD2A mRNA level and a larger proportion of high KDM2A expression (all *P* < 0.05). High KDM2A mRNA expression was more likely to occur in ccRCC tissues with tumor size > 7 cm (*P* = 0.005) and T3-T4 stage (*P* = 0.047). Knockdown of KDM2A significantly suppressed the proliferation and invasion, and promoted the apoptosis of ccRCC cells (all *P* < 0.05). Moreover, Kaplan–Meier survival analysis revealed that higher level of KDM2A expression in ccRCC patients was associated with lower survival rate (*P* = 0.004).

**Conclusions:**

Our findings demonstrated a vital role of KDM2A in the pathogenesis of ccRCC, which provides a new perspective to understand the underlying molecular mechanisms in ccRCC.

## Background

Kidney cancer is a common malignancy of urinary system, whereas renal cell carcinoma (RCC) is the most common kidney neoplasm and contains a cluster of heterogeneous tumors which originate from the renal tubular epithelial cells [1, 2]. At present, the accepted treatment of RCC is mainly surgical resection. Clear cell renal cell carcinoma (ccRCC) is the most frequent subtype of RCC, which nearly accounts for about 75% of RCC [3]. It derives from the proximal tubular epithelium and is characterized by the worst clinical process and prognosis among the classes of RCC. However, the genetic and epigenetic background of changes that occur during the initiation and development of ccRCC remains poorly understood [4]. Therefore, to illuminate the molecular mechanisms underlying ccRCC tumorigenesis and progression so as to identify novel biological makers and targets for effective treatments for ccRCC is of great significance.

Covalent histone modifications play a critical part in the regulation of chromatin dynamics and functions [5]. Histone methylation is an important form of epigenetic modification and occurs on both lysine and arginine residues. Through the methylation of different histone sites, gene expressions can be regulated by influencing the activation and inhibition of transcription [6]. The constant level of covalent histone methylation is under the control of histone methyltransferases and demethylases [7]. The lysine-specific demethylase 2A gene (KDM2A), found on chromosome 11q13.2, is a member of the KDM histone lysine demethylase family, which exhibits specificity for removal of methyl groups of histone H3K36 by binding directly to CpG islands in gene promoters [7]. The dysfunction of KDM2A has been reported in various cancers, and its loss-of-function mouse mutants are embryonically lethal [7]. However, KDM2A has a complex and tissue-specific role in tumorigenesis and tumor progression. An increasing number of studies have described that KDM2A exerts an oncogenic role in a wide range of tumor types, including breast cancer, gastric cancer and lung cancer, and can enhance the growth and motility of cancer cells to promote tumor progression [8–12]. On the contrary, Frescas et al. found that KDM2A expression was often decreased in prostate cancer compared with normal prostate tissue [13]. However, its expression pattern and biological function in ccRCC have not yet been elucidated.

The current study aimed to investigate the expression pattern of KDM2A in vitro and in vivo, and determine the biological impact of KMD2A in ccRCC cell line. Then, the prognostic role of KDM2A in ccRCC was evaluated on the basis of survival data from TCGA. All together may provide novel insight into the development of therapeutic strategies for ccRCC.

## Methods

### Patients and samples

Fifty patients with ccRCC who underwent radical nephrectomy at the First Hospital of China Medical University from January 2018 to October 2018 were enrolled in the study. ccRCC tissues and corresponding para cancer tissues were obtained from renal operation and immediately stored at − 80 °C. The para cancer tissues were taken from a location 5 cm away from the tumor. For some patients with a large tumor with the remaining para cancer tissues being less than 5 cm, the renal tissues more than 3 cm away from the tumor were taken and pathologically proven to be cancer-free. No patients had received any preoperative adjuvant therapy, such as radiotherapy, chemotherapy, immunotherapy, targeted therapy, interventional embolization, and so on, or had a history of other malignant tumors. Demographic and clinical data of ccRCC patients are summarized in Table 1. This study was approved by the Ethics Committee of the First Hospital of China Medical University (Shenyang, China). Written informed consent was obtained from each subject.

**Table 1:** Baseline characteristics of ccRCC patients (n = 50)

Parameters	ccRCC
*Gender, n (%)*	
Male	29 (58%)
Female	21 (42%)
Age, years	58.78 ± 8.85
< 60, n (%)	28 (56%)
≥ 60, n (%)	22 (44%)
*Location, n (%)*	
Left	22 (44%)
Right	28 (56%)
BMI (kg/m^2^)	24.79 ± 3.22
< 24, n (%)	20 (40%)
≥ 24, n (%)	30 (60%)
Tumor size(cm)	6.20 ± 2.38
≤ 7, n (%)	35 (70%)
> 7, n (%)	15 (30%)
*Smoking, n (%)*	
No	39 (78%)
Yes	11 (22%)
*Thrombus of renal vein, n (%)*	
No	46 (92%)
Yes	4 (8%)
*TNM stage, n (%)*	
T_1_	27 (54%)
T_2_	11 (22%)
T_3_	10 (20%)
T_4_	2 (4%)
T_1_–T_2_	38 (76%)
T_3_–T_4_	12 (24%)
*ISUP grade, n (%)*	
1	4 (8%)
2	31 (62%)
3	8 (16%)
4	7 (14%)
1–2	35 (70%)
3–4	15 (30%)
*Symptoms, n (%)*	
No	32 (64%)
Yes	18 (36%)
*Hypertension, n (%)*	
No	29 (58%)
Yes	21 (42%)

### Cell culture and transfection

Human ccRCC cells (786-O) and Human kidney proximal tubular cells (HK-2) were purchased from the Cell Bank of the Chinese Academy of Sciences (Shanghai, China). 786-O cells were cultured in RPMI 1640 medium (HyClone, Logan, UT, USA) containing 10% fetal bovine serum (FBS) (HyClone, Logan, UT, USA). HK-2 cells were cultured in DMEM/F12 medium (HyClone, Logan, UT, USA) containing 10% FBS. The cells were cultured at 37 °C in an incubator with 5% CO2. si-KMD2A and si-NC (GenePharma, Shanghai, China) were transfected into 786-O cells using Lipofectamine 2000 transfection reagent (Invitrogen, Grand Island, NY, USA) following the manufacturer’s protocol.

### Western blotting assay

The cells were lysed in cold RIPA Lysis Buffer (Beyotime, Shanghai, China), and the protein concentration was determined using a BCA Protein Assay Kit (Beyotime, Shanghai, China). After boiling in loading buffer at 95 °C for 5 min, 40 μg per lane of proteins were separated by 8% SDS-PAGE and then transferred to polyvinylidene difluoride membranes (Millipore, Billerica, MA, USA). The membranes were blocked using 5% (W/V) non-fat milk in Tris-buffered saline containing 0.1% (V/V) Tween-20 at room temperature for 1 h and then incubated overnight at 4 °C with the primary antibodies: rabbit anti-human KDM2A (1:1,000; Abcam, Cambridge, MA, USA) and rabbit anti-human β-actin (1:3,000; Proteintech, Rosemont, IL, USA). After several washings, the membranes were then incubated with HRP-conjugated affinipure goat anti-rabbit secondary antibody (1:5,000; Proteintech, Rosemont, IL, USA) for 45 min at 37 °C. The protein bands were visualized using a super ECL plus kit (US Everbright, Suzhou, China). The levels of protein expression were evaluated by ImageJ software (version 1.41; National Institutes of Health, Bethesda, MD, USA).

### qRT-PCR

All the tissues were used to determine KDM2A mRNA level. Total mRNAs of ccRCC and paracancer tissues were extracted using TRIzol reagent (Invitrogen, Carlsbad, CA, USA.) following the manufacturer’s protocols. Reverse-transcribed cDNA synthesis was performed with BioTeke Super RT kit (BioTeke, Beijing, China). Real-time PCR was conducted using 2 × Power Taq PCR MasterMix (BioTeke, Beijing, China) and SYBR Green (BioTeke, Beijing, China) according to the manufacturer’s instructions. β-actin acted as an internal control. The primers were synthesized as follows: KDM2A Forward 5′-GGCAGTAGGAATCAAGGACC-3′, Reverse 5′-ACCCGACAGCAGTGAGTAGA-3′; β-actin: Forward 5′-CACTGTGCCCATCTACGAGG-3′, Reverse 5′-TAATGTCACGCACGATTTCC-3′. PCR conditions were as follows: initial denaturation at 94 °C for 5 min, followed by 40 cycles of 94 °C for 15 s, 60 °C for 20 s, and 72 °C for 30 s. Each reaction was set up 3 times and the expression level of KDM2A was quantified using the 2^−△△Ct^ method.

### Immunohistochemistry (IHC)

All the tissues were fixed in 4% paraformaldehyde, embedded in paraffin, and sectioned at 4 μm. Then, the slides were treated with xylene to remove the paraffin, followed by hydration with ethanol and the addition of EDTA for antigen retrieval. Endogenous peroxidase blocker solution was added to incubate for 10 min, and the sections were rinsed with phosphate buffer saline (PBS) 3 times. To avoid nonspecific binding, normal goat serum was added to block tissue collagen for 10 min. Sections were incubated with KDM2A monoclonal antibody (1:250; Abcam, Cambridge, MA, USA) for 1 h at room temperature. After washing 3 times with PBS, the sections were incubated with biotinylated secondary goat anti-rabbit antibody, and then with streptavidin–biotin peroxidase for 10 min each. Finally, the diaminobenzidine solution was used to stain the sections, which were then counterstained with hematoxylin.

The KDM2A IHC score was determined by both the intensity and percentage of cellular staining. The staining intensity was divided into scores of 0 (negative), 1 (mild), 2 (moderate), 3 (strong), and the percentage was classified as 1 (0–25%), 2 (26–50%), 3 (51–75%), and 4 (> 75%). The intensity and percentage were multiplied to calculate the total IHC staining score, which was assigned as negative staining (−, 0), mild staining (+, 1–4), moderate staining (++, 5–8), severe staining (+++, 9–12). An IHC score ≥ 5 was defined as high expression, while a score of less than 5 was defined as low expression. Two independent observers were employed to assess and examine immunostaining.

### Cell counting kit-8 (CCK-8) assay

Cells were seeded in 96-well plates at 3,000 cells per well and cultured for 24, 48 and 72 h. Then, 10 µl of CCK-8 (Dojindo Molecular Technologies, Kumamoto, Japan) was added and subsequently incubated for 1 h at 37 °C. The absorbance (optical density value) was measured at 450 nm.

### Transwell assay

The cells were seeded at a density of 1 × 10^4^ cells/well into the upper transwell chamber (Corning, NY, USA), which contained 200 µl serum-free medium, and 800 µl of the medium containing 20% FBS was added to the lower chamber. Following 48 h of incubation at 37 °C, the cells remaining on the upper membrane were removed with a sterile cotton swab. The chambers were fixed at room temperature with 4% paraformaldehyde for 20 min, and then stained with 0.1% crystal violet (Solarbio, Beijing, China) for 30 min. The numbers of stained cells were counted in five different fields using an inverted microscope at a magnification of × 200.

### Cell apoptosis measured by flow cytometry

The cell apoptosis was assessed using an Annexin V-fluorescein isothiocyanate (FITC) /propidium iodide (PI) Apoptosis Detection Kit (Wanleibio, Shenyang, China). The cells were rinsed twice with sterile phosphate buffer saline and resuspended in 500 ul binding buffer. Subsequently, stained the cells with 5 μl Annexin V-FITC and 10 μl PI in the darkness for 15 min. Analysis was performed using a NovoCyte flow cytometer (ACEA Biosciences, San Diego, CA, USA).

### Kaplan–Meier curve

Survival data from TCGA was obtained to clarify the prognostic value of KDM2A in ccRCC. They were submitted to OncoLnc (http://www.oncolnc.org/) and Kaplan–Meier curve was plotted for prognostic analysis.

### Statistical analysis

Statistical analysis was performed using SPSS version 23 (IBM, Armonk, NY, USA). Quantitative data were expressed as the mean ± standard deviation (SD), and counting data were represented by the number and rate. The Kolmogorov–Smirnov test was performed to test whether the data were normally distributed. χ^2^ test or Fisher’s exact test was performed to compare the categorical variables. Differences between groups were calculated by independent sample t-test or one-way ANOVA. *P* < 0.05 was considered to indicate a statistically significant difference.

## Results

### Baseline characteristics of ccRCC patients

The patients’ baseline characteristics are presented in Table 1. There were 29 (58%) male and 21 (42%) female cases with an average age of 58.78 years. TNM stage T1-T2 and T3-T4 accounted for 38 (76%) and 12 (24%) of all the patients, respectively. As for the International Society of Urological Pathology (ISUP) grade, 35 patients (70%) were classified as grade 1–2, while 15 patients (30%) were classified as grade 3–4. Moreover, most patients had no clinical symptoms.

### KDM2A expression in ccRCC cell lines and tissues

**Fig. 1:**
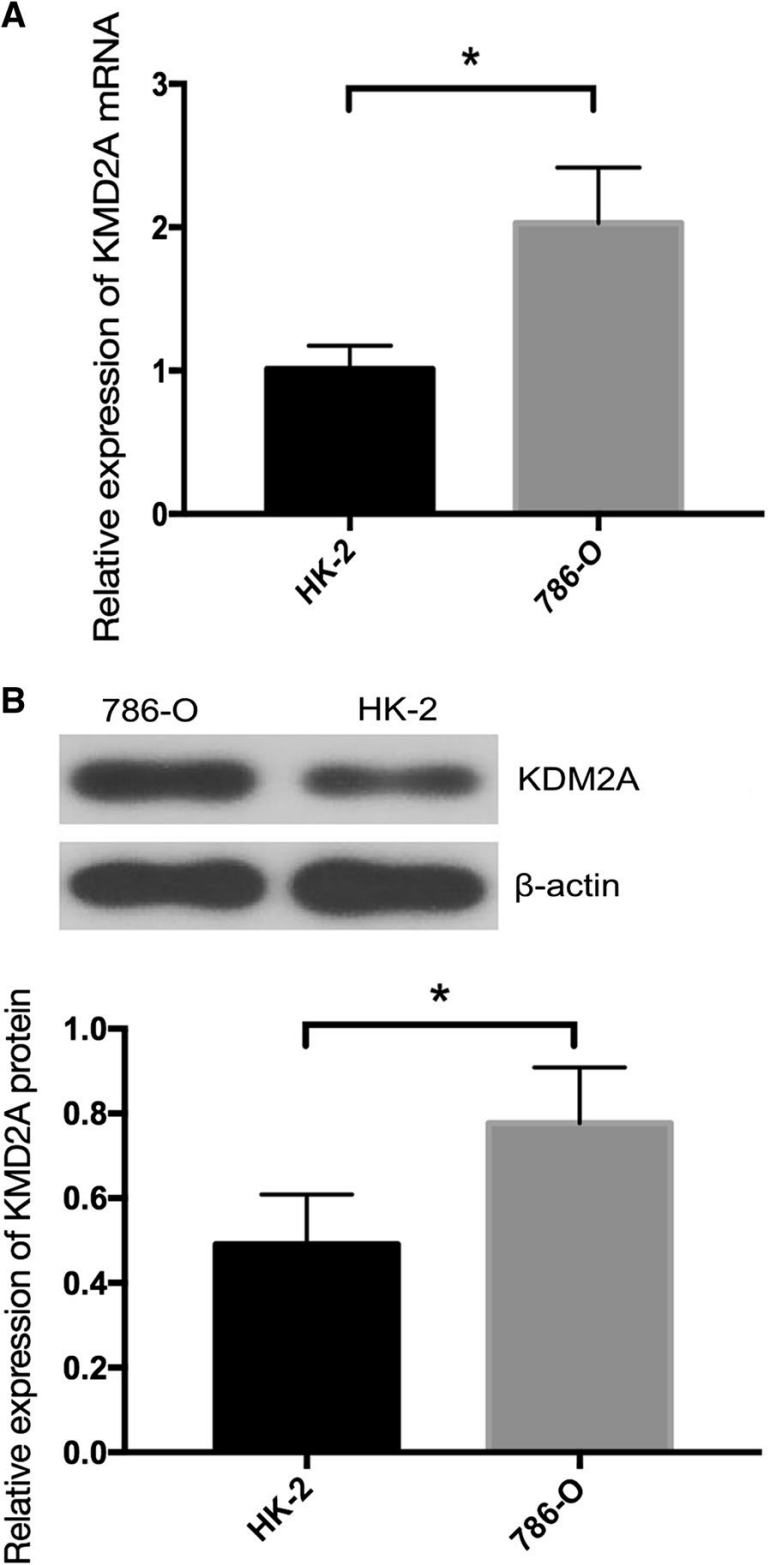
KDM2A expression in cell lines. **a** qRT-PCR analysis and **b** western blot analysis indicated that KDM2A mRNA and protein expression levels were higher in 786-O cells compared with HK-2cells. *P < 0.05

As shown in Fig. 1, qRT-PCR and western blot were used to exam KDM2A expression in cell lines. The mRNA and protein expression of KDM2A were both significantly upregulated in 786-O cells in comparison with HK-2 cells (*P* < 0.05).

**Fig. 2:**
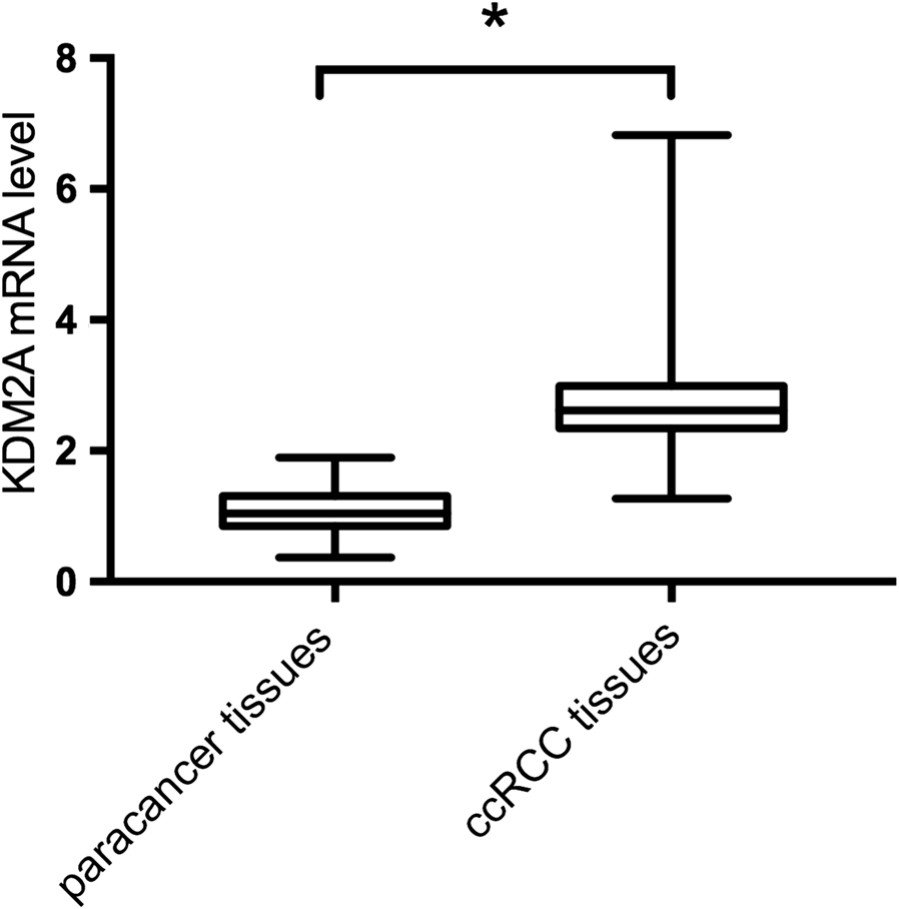
KDM2A mRNA expression was detected by qRT-PCR and shown to be about 2.59-fold higher in ccRCC samples than in para cancer samples. *P < 0.05

**Fig. 3:**
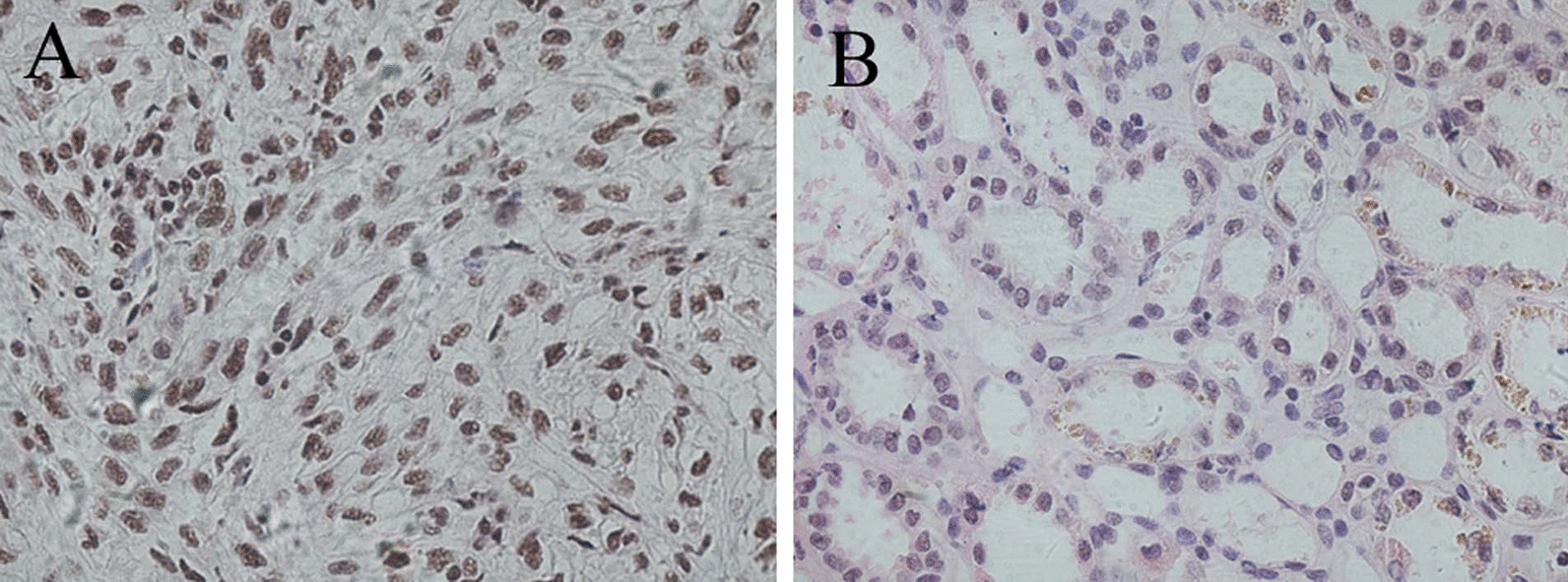
Immunohistochemical staining for KDM2A protein expression in ccRCC and para cancer tissues (Magnification, × 400). **a** High expression of KDM2A in ccRCC tissues. **b** Low expression of KDM2A in para cancer tissues

KDM2A mRNA expression was around 2.59-fold higher in ccRCC samples than that in para cancer samples with a significant difference (*P* < 0.05; Fig. 2). According to the median ratio of KDM2A mRNA level (2.61) in tumor tissues, ccRCC patients were classified into the high and low KDM2A mRNA expression groups. IHC analysis revealed that KDM2A protein was predominantly located in the cell nucleus and ccRCC samples had significantly higher KDM2A expression levels than para cancer tissues (Fig. 3). In addition, based on IHC results, all the ccRCC samples were immunopositive, 90% of which showed a high expression of KDM2A protein, which was significantly higher than that in para cancer tissues (Table 2).

**Table 2:** IHC analysis displayed the expression of KDM2A protein in ccRCC and para cancer tissues

Group	(−)	(+)	(++)	(+++)	*P*	Low, n (%)	High, n (%)	*P*
Para cancer tissues	8 (16%)	17 (34%)	14 (28%)	11 (22%)	Reference	25 (50%)	25 (50%)	Reference
ccRCC tissues	0 (0%)	5 (10%)	19 (38%)	26 (52%)	< 0.001	5 (10%)	45 (90%)	< 0.001

Furtherly, we analyzed the correlations of KDM2A mRNA and protein expression with clinical features in ccRCC and found that the high expression of KDM2A mRNA was more likely to occur in ccRCC tissues with tumor size > 7 cm (*P* = 0.005) and T3-T4 stage (*P* = 0.047) (Table 3). However, there was no significant association between KDM2A protein expression and clinical features in ccRCC (Table 4).

**Table 3:** Association between KDM2A mRNA expression and clinical features in ccRCC

Parameters	n	KDM2A mRNA expression in ccRCC		*P*
		Low, n (%)	High, n (%)	
*Gender*				
Male	29	12 (41.4%)	17 (58.6%)	0.152
Female	21	13 (61.9%)	8 (38.1%)	
*Age, years*				
< 60	28	13 (46.4%)	15 (53.6%)	0.569
≥ 60	22	12 (54.5%)	10(45.5%)	
*Location*				
Left	22	12 (54.5%)	10 (45.5%)	0.569
Right	28	13 (46.4%)	15 (53.6%)	
*BMI (kg/m*^*2*^*)*				
< 24	20	7 (35.0%)	13 (65.0%)	0.083
≥ 24	30	18(60.0%)	12 (40.0%)	
*Tumor size (cm)*				
≤ 7	35	22 (62.9%)	13(37.1%)	0.005
> 7	15	3 (20.0%)	12 (80.0%)	
*Smoking*				
No	39	20 (51.3%)	19 (48.7%)	0.733
Yes	11	5 (45.5%)	6 (54.5%)	
*Thrombus of renal vein*				
No	46	24 (52.2%)	22 (47.8%)	0.602
Yes	4	1 (25.0%)	3 (75.0%)	
*TNM stage*				
T_1_–T_2_	38	22 (57.9%)	16 (42.1%)	0.047
T_3_–T_4_	12	3 (25.0%)	9 (75.0%)	
*ISUP grade*				
1–2	35	19(54.3%)	16 (45.7%)	0.355
3–4	15	6 (40.0%)	9 (60.0%)	
*Symptoms*				
No	32	15 (46.9%)	17 (53.1%)	0.556
Yes	18	10 (55.6%)	8 (44.4%)	
*Hypertension*				
No	29	16 (55.2%)	13 (44.8%)	0.390
Yes	21	9 (42.9%)	12 (57.1%)

**Table 4:** Association between KDM2A protein expression and clinical features in ccRCC

Parameters	n	KDM2A mRNA expression in ccRCC		*P*
		Low, n (%)	High, n (%)	
*Gender*				
Male	29	1 (3.4%)	28 (96.6%)	0.181
Female	21	4 (19.0%)	17 (81.0%)	
*Age, years*				
< 60	28	3 (10.7%)	25 (89.3%)	1.000
≥ 60	22	2 (9.1%)	20 (90.9%)	
*Location*				
Left	22	2 (9.1%)	20 (90.9%)	1.000
Right	28	3 (10.7%)	25 (89.3%)	
*BMI (kg/m*^*2*^*)*				
< 24	20	0 (0%)	20 (100%)	0.149
≥ 24	30	5 (16.7%)	25 (83.3%)	
*Tumor size (cm)*				
≤ 7	35	5 (14.3%)	30 (85.7%)	0.304
> 7	15	0 (0%)	15 (100%)	
*Smoking*				
No	39	5 (12.8%)	34 (87.2%)	0.495
Yes	11	0 (0%)	11 (100%)	
*Thrombus of renal vein*				
No	46	5 (10.9%)	41 (89.1%)	1.000
Yes	4	0 (0%)	4 (100%)	
*TNM stage*				
T_1_–T_2_	38	5 (13.2%)	33 (86.8%)	0.440
T_3_–T_4_	12	0 (0%)	12 (100%)	
*ISUP grade*				
1–2	35	4 (11.4%)	31 (88.6%)	1.000
3–4	15	1 (6.7%)	14 (93.3%)	
*Symptoms*				
No	32	4 (12.5%)	28 (87.5%)	0.768
Yes	18	1 (5.6%)	17 (94.4%)	
*Hypertension*				
No	29	4 (13.8%)	25 (86.2%)	0.567
Yes	21	1 (4.8%)	20 (95.2%)	

### Knockdown of KDM2A suppresses the proliferation and invasion, and promotes the apoptosis of ccRCC cells

**Fig. 4:**
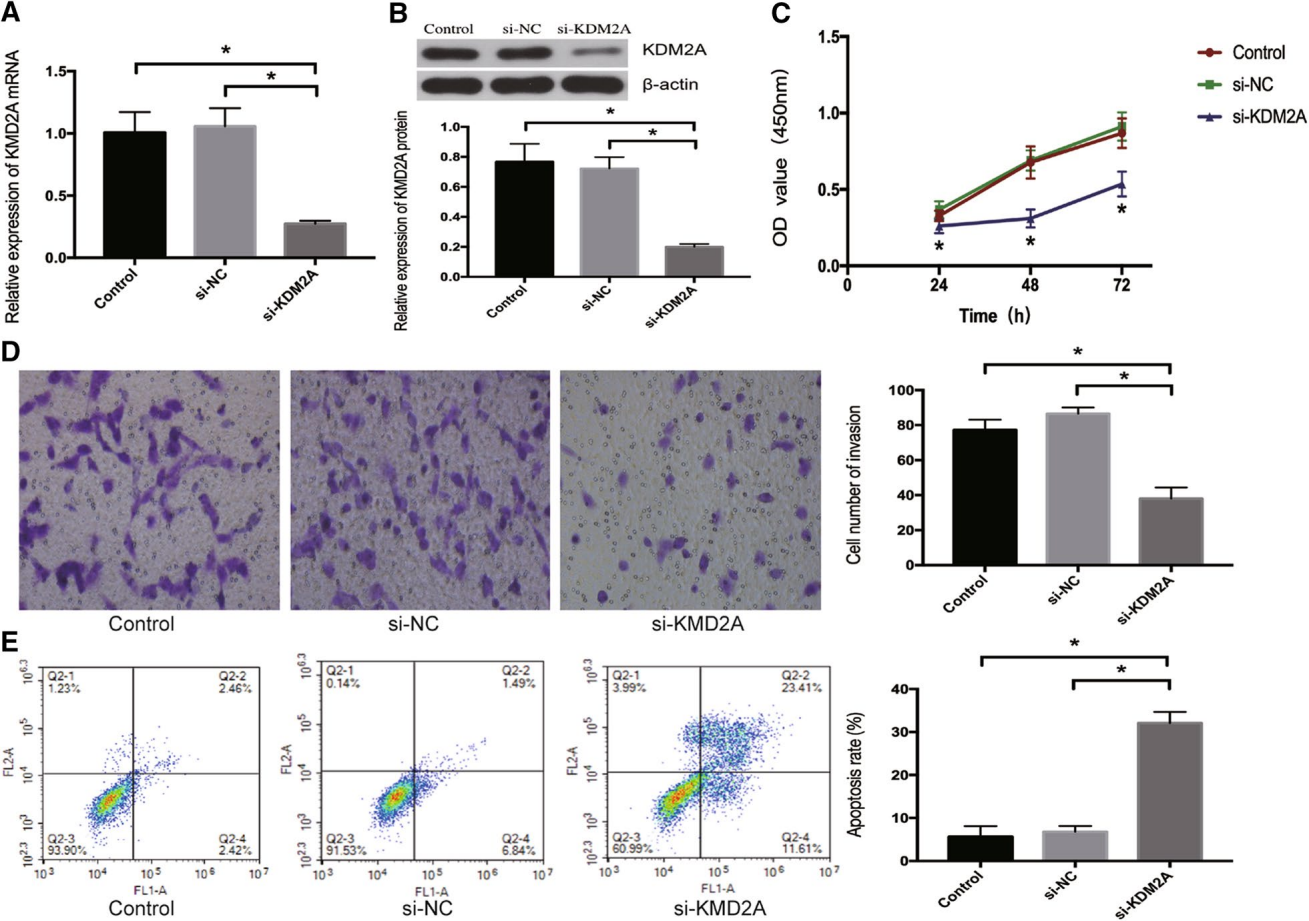
Knockdown of KDM2A suppresses the proliferation and invasion, and promotes the apoptosis of ccRCC cells. **a** qRT-PCR analysis and **b** western blot analysis showed that KDM2A mRNA and protein expression levels in 786-O cells transfection with si-KDM2A were notably downregulated than control and si-NC groups. **c** The proliferative ability of 786-O cells transfected with si-KDM2A was determined by CCK‑8 assay. **d** Representative image of transwell assay, quantitative measurement of the number of transmembrane cells indicates that knockdown of KDM2A reduces cellular invasion (Magnification, × 200). **e** The results of flow cytometry demonstrated that 786-O cells transfected with si-KDM2A had a higher apoptosis percentage (32.10 ± 2.60%) than control (5.66 ± 2.46%) and si-NC (6.83 ± 1.31%) group. *P < 0.05

A loss-of function experiment was performed to determine the biological role of KDM2A in ccRCC. The efficiency of knockdown was confirmed by qRT-PCR and western blot analysis. The results showed that transfection with si-KDM2A notably decreased the mRNA and protein expression of KDM2A in 786-O cells compared to control and si-NC groups (Fig. 4a, b). CCK‑8 and transwell assays revealed that si-KDM2A significantly inhibited the proliferative and invasive ability of 786-O cells when compared with control and si-NC groups, respectively (*P* < 0.05) (Fig. 4c, d). The results of flow cytometry demonstrated that 786-O cells transfected with si-KDM2A had an apoptosis percentage of 32.10 ± 2.60%, which was significantly higher than control and si-NC group with cell apoptosis rate of 5.66 ± 2.46% and 6.83 ± 1.31%, respectively (*P* < 0.05) (Fig. 4e).

### The prognostic value of KDM2A in ccRCC patients

**Fig. 5:**
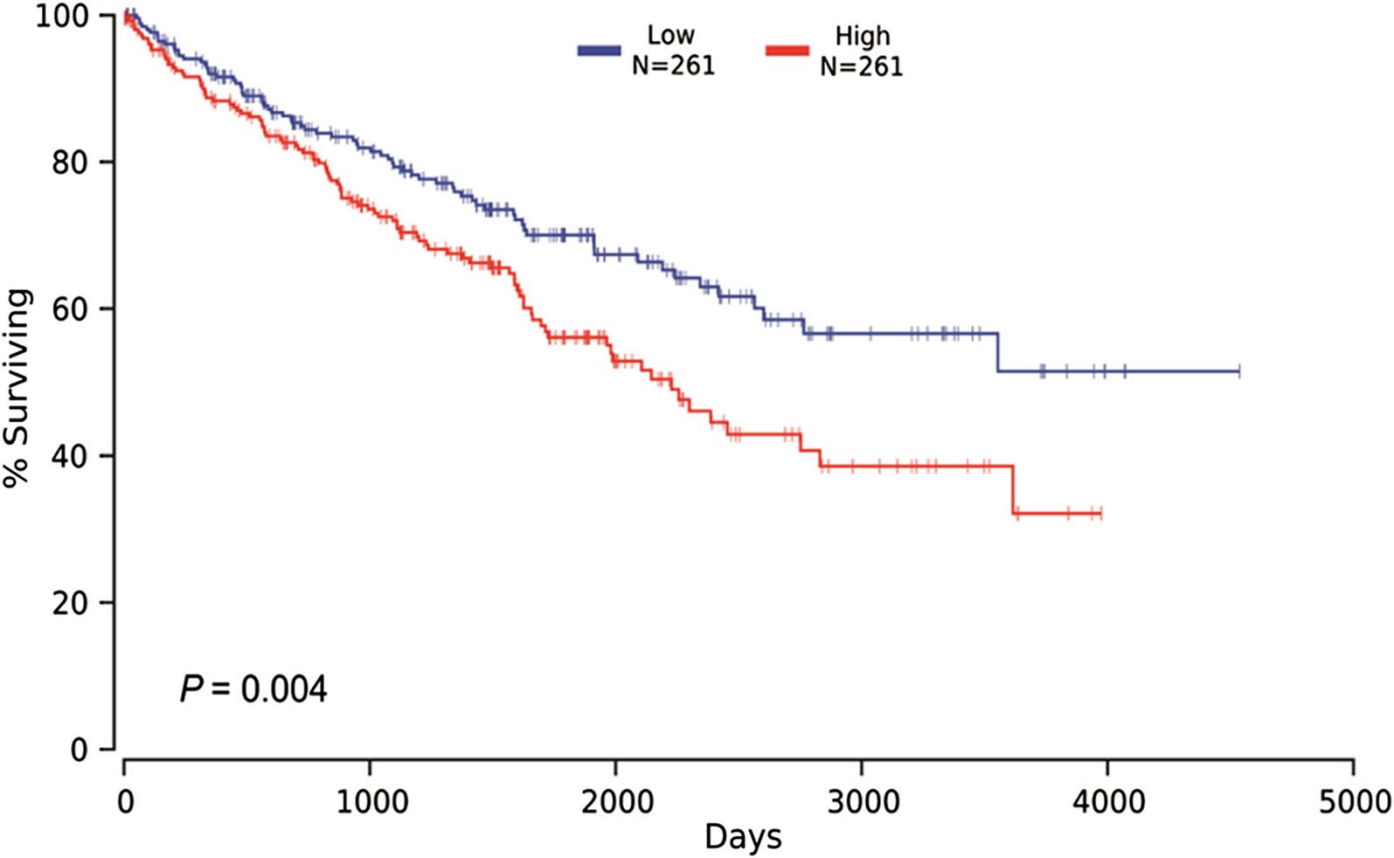
Kaplan–Meier survival curve was performed to assess the prognostic value of KDM2A in ccRCC

Kaplan–Meier survival analysis suggested that higher expression of KDM2A in ccRCC patients was related to lower survival rate (*P* = 0.004, Fig. 5).

## Discussion

In the current study, we are the first, to our knowledge, to make a comprehensive evaluation of KDM2A expression pattern and regulatory effect in ccRCC. We found that KDM2A level was upregulated in ccRCC cell line and tissues, and knockdown of KDM2A suppressed ccRCC cells to proliferate and invade and promoted their apoptosis. Moreover, high KDM2A mRNA expression in ccRCC was associated with large tumor size, advanced TNM stage and a poor survival prognosis. Our research may provide a theoretical basis for further pathological studies and the improvement of novel therapeutic approaches for ccRCC patients.

The occurrence and development of ccRCC is a complicated multi-step biological process, which may involve the instability of the genome, the gradual accumulation of gene mutations, epigenetic mechanism alterations, and abnormal gene expression [14, 15]. Histone lysine methylation was regarded as a central modification for the post-transcriptional regulation of chromosome structure and DNA replication, repair, and transcription procedure [16], and was relevant to the activation or silencing of gene expression [17]. KDM2A can specifically catalyze the demethylation of histone H3K36, which is a conserved epigenetic marker influencing gene transcription, alternative splicing, and DNA repair [18, 19]. During cell mitosis, KDM2A plays a role in maintaining genomic stability and centromeric integrity [13]. Meanwhile, the overexpression of KDM2A might antagonize the senescence of embryonic fibroblasts and promote somatic reprogramming [18]. The deletion of KDM2A could also inhibit the proliferation of stem cells from apical papilla [16]. In addition, Xu et al. found that KDM2A might be an important regulator of cell proliferation and cell cycle via impacting TGF-β signaling pathway [20].

Emerging studies have shown that the level of KDM2A expression is up-regulated in a variety of tumor cells and affects the biological behavior of tumor cells. In the present study, KDM2A expression in ccRCC was evaluated using multiple methods, which have never been reported before. Our results found that the mRNA and protein expression levels of KDM2A in ccRCC cells and tissues were significantly upregulated. Moreover, an elevated percentage of high KDM2A mRNA expression was markedly demonstrated in ccRCC tissues with tumor size > 7 cm and T3-T4 stage. Furthermore, we investigated the functional role of KDM2A in ccRCC cells, revealing that KDM2A silencing significantly suppressed the proliferation and invasion, and induced the apoptosis of 786-O cells. Based on the survival data from TCGA, it was demonstrated that ccRCC individuals with high KDM2A level would have a relatively worse survival prognosis. Our results indicated that KDM2A may play a key role in the initiation and progression of ccRCC, and could serve as a diagnostic and prognostic biomarker for this disease.

Similarly, gastric cancer tissues have been found to have an increased level of KDM2A expression and forced expression of KDM2A promoted cell growth and migration [8]. In breast cancer, KDM2A was found to be highly expressed and significantly correlated with shortened survival of breast cancer patients [11, 21]. In non-small cell lung cancer (NSCLC), Wagner et al. demonstrated that KDM2A was overexpressed and could promote the proliferation and metastasis of NSCLC cells by regulating the activity of ERK1/2 pathway, which was related to a poor prognosis of NSCLC patients [9]. An up-regulated expression of KDM2A was also shown to play a critical role in the onset and progression of cervical cancer and promote the proliferation and invasion of cervical cancer cells [22, 23]. However, Frescas et al. revealed a lower level of KDM2A expression in prostate carcinomas compared to normal prostate tissue [13]. These diverse observations may be attributed to the heterogeneity of disease and a variety of sample sizes.

Some limitations existed in our study. Firstly, there was a lack of comparison with normal kidneys. Secondly, due to the lack of prognostic information in our ccRCC patients, the prognostic results only relied on the data from TCGA. In addition, we did not verify our work in animals. Further studies that include a larger number of subjects with detailed clinical information are necessary to confirm our findings.

## Conclusions

In summary, we observed the high expression of KDM2A in ccRCC cell line and tissues. High KDM2A mRNA expression was positively correlated with tumor size and TNM stage, and had an adverse effect on the overall survival of ccRCC patients. Additionally, the knockdown of KDM2A decreased cell proliferation and invasion, and increased the apoptosis in ccRCC. Our results unravel a new regulatory mechanism involved in ccRCC pathogenesis and progression, and KDM2A may be a potential marker for the diagnosis and prognosis of ccRCC patients.

## Data Availability

The datasets used and/or analyzed during the current study are available from the corresponding author on reasonable request.

## Abbreviations

KDM2A: Lysine-specific demethylase 2A
ccRCC: Clear cell renal cell carcinoma
RCC: Renal cell carcinoma
FBS: Fetal bovine serum
IHC: Immunohistochemistry
PBS: Phosphate buffer saline
CCK-8: Cell counting kit-8
FITC: Fluorescein isothiocyanate
PI: Propidium iodide
SD: Standard deviation
ISUP: International Society of Urological Pathology
NSCLC: Non-small cell lung cancer
